# Rapid Manufacturing of Glass‐Based Digital Nucleic Acid Amplification Chips by Ultrafast Bessel Pulses

**DOI:** 10.1002/smsc.202300166

**Published:** 2023-12-28

**Authors:** Jiawei Zhang, Kotaro Obata, Kazunari Ozasa, Takanori Uzawa, Yoshihiro Ito, Koji Sugioka

**Affiliations:** ^1^ RIKEN Center for Advanced Photonics 2‐1 Hirosawa Wako Saitama 351‐0198 Japan; ^2^ RIKEN Center for Emergent Matter Science 2‐1 Hirosawa Wako Saitama 351‐0198 Japan

**Keywords:** digital NAAT, glass micromaching, ultrafast laser

## Abstract

Advanced nucleic acid amplification techniques require separating test samples into numerous units (digitalization), which enables higher sensitivity and accuracy than traditional methods. Reagent partition tools are commercially available, but are usually complex and expensive, hindering mass adoption. Herein, the fabrication of a large‐scale micro‐through‐hole array on glass substrates for an advanced digital nucleic acid amplification technique (NAAT) is demonstrated. To meet the requirement of hole quantities (over tens of thousands) for valid nucleic acid statistics, ultrafast Bessel pulses with continuous translation of the glass substrate are applied. A single‐shot Bessel pulse can lead to a single hole, and 100 holes can be produced per second (scalable to thousands per second). Experiments using the fabricated chips show that the performance meets the NAAT requirements. This work provides a highly efficient and low‐cost scheme for reagent partitioning that promotes the wide accessibility of advanced digital NAATs as well as a variety of other applications.

## Graphical abstract

Glass based large‐scale micro‐through hole array is fabricated using an ultrafast laser. Picosecond Bessel pulses are incident on a fast‐moving glass substrate to create an array of through glass modifications, which is then treated with chemicals to form holes with larger dimensions. Biological reagents are loaded into the array holes for digital nucleic acid amplification.

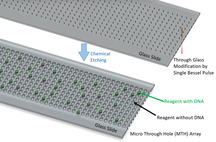

## 1 Introduction

Nucleic acid amplification technique (NAAT) is a highly valuable method for drastically amplifying and detecting trace numbers of targeted DNA or RNA molecules in biological samples and plays a key role in the field of modern molecular biology.^[^ ^1^, ^2^, ^3^ ^]^ NAAT has a wide range of applications in clinical diagnostics, environmental pathogen monitoring, genetic engineering, and DNA sequencing. Various NAATs are currently available. One of the most well‐known and widely used NAATs is the polymerase chain reaction (PCR), which is carried out routinely around the world. Other techniques include loop‐mediated isothermal amplification and recombinase polymerase amplification (RPA), which are promising alternatives to PCR that do not require thermal cycling.^[^ ^4^ ^]^ Current mainstream NAATs can in general be categorized as first‐generation analog schemes, in which target DNA (RNA) samples are mixed with a suitable reagent in a single macroscopic test tube to be amplified. During the amplification process, fluorescent molecules are released, which generate optical signals that can be recorded by photodetectors. The next generation of NAATs is expected to be based on a digital scheme, in which a mixture of DNA (RNA) samples and a reagent is partitioned into a large number (usually tens of thousands) of independent units, so that the majority of these units contain either no DNA (RNA) or just a single molecule.^[^ ^5^, ^6^ ^]^ During amplification, fluorescence signals are only obtained from the units that initially contain DNA molecules. Therefore, by counting the number of such units after amplification, the concentration of DNA molecules can be accurately estimated. Digital NAAT (such as digital PCR) offers several important advantages, including higher precision and sensitivity, absolute quantification without the need for a standard curve, high tolerance to inhibitors, and the ability to test multiple targets simultaneously.^[^ ^5^ ^]^ However, widespread use of digital NAAT is nowadays still limited, mainly due to the need for complex equipment and reagent partition steps during sample preparation, which usually result in low throughput and high cost. Currently, there are two major methods for reagent partitioning: microfluidic‐based and plate‐based. In the former, microfluidic devices are used to generate microscopic reagent droplets in oil. This requires the use of multiple pumping systems and usually exhibits limited sample preparation efficiency.^[^ ^7^ ^]^ On the other hand, the plate‐based method uses a large array of microholes which enables easy and fast reagent partitioning without the need for pumping systems. To date, fabrication of microhole arrays for digital NAAT has been demonstrated using silicon and polymer substrates,^[^ ^5^, ^8^ ^]^ both of which have advantages and disadvantages.^[^ ^9^ ^]^ Although silicon‐based microhole array chips have been commercialized for scientific research for years,^[^ ^10^ ^]^ their widespread applications in clinical diagnosis is limited by the relatively high cost of silicon wafers and the complicated manufacturing process. Polymer‐based materials usually have hydrophobic surfaces, which makes it difficult to partition reagent into microholes, and the low thermal conductivity of polymers makes them inferior for NAATs like PCR, which requires a fast thermal cycling process.

In contrast, glass is a superior material platform that has been used in biomedicine for decades. Its merits include good optical transparency, low fluorescence, high thermal stability, autoclavability, and flexible surface modification.^[^ ^11^, ^12^ ^]^ For digital NAAT, specifically, glass‐based micro‐array chips offer clear cost advantages over silicon and compared to polymer‐based chips, allow easier reagent partitioning due to the hydrophilicity of glass and higher applicability to the thermal cycling process due to larger thermal conductivity, as required by PCR process. However, due to the hardness and brittleness of glass, efficient fabrication of large‐scale arrays of compact and crack‐free microholes with dimensions of several hundred micrometers in diameter and depth in glass has been technically challenging.^[^ ^13^ ^]^ For example, although mechanical drilling is a simple and relatively cost‐effective method, it is prone to cracking because of the thrust force of the drill acting on the bottom of the glass substrate. More seriously, it spent lots of time to drill numerous holes due to typical drilling feed rate of 5 μm s^−1^. Chemical etching with photolithography requires multiple steps with consumption of significant amounts of chemicals. Beyond these traditional methods, the recent development of ultrafast laser‐induced selective etching (ULISE) has increasingly become a more attractive option for producing such arrays because it allows highly flexible fabrication of crack‐free and high‐precision microholes.^[^ ^14^, ^15^, ^16^ ^]^ ULISE generally involves local laser modification of glass followed by chemical treatment with hydrofluoric acid (HF) or potassium hydroxide (KOH) to preferentially etch away the laser modified regions (LMRs) due to the much higher etching rate than nonmodified regions.^[^ ^17^, ^18^, ^19^, ^20^ ^]^ ULISE thus enables fabrication of high‐quality, high‐aspect‐ratio holes in glass substrates. In the present study, we propose a scheme to demonstrate an efficient method of writing 20 000 micro‐through‐holes (MTHs) in a glass slide by ULISE using a Bessel beam for the purpose of digital NAAT. The entire process is shown to require only 300 s. Bessel beam is a nondiffractive beam and can remain focused at a long range, typically longer than several mm, so that it is beneficial to create high‐aspect structures inside the materials. However, a significant portion of the laser energy does not contribute to the processing, because the thickness of substrates processed is thinner than the depth of focus. In this study, for more efficient utilization of laser energy, the Bessel beam was tailored to make the depth of focus almost same as the thickness of the glass substrate. Note that although ULISE using a Bessel beam for glass processing has been demonstrated in previous works, they primarily focus on through glass vias (TGVs) creation for the purpose of semiconductor chip packaging.^[^ ^21^, ^22^ ^]^ Importantly, in contrast to fabrication of TGVs for semiconductor packaging, dNAAT requires enormous number of holes (tens or even hundreds of thousands of units) for accurate analysis of DNA concentration, which makes laser processing inefficient. Additionally, dimensions of holes for each application are different. Specifically, the dimension necessary for dNAAT is much larger than that for TGVs, because each hole in the dNAAT chip should accommodate several nL of sample volume for the analysis. Importantly, not only the ability to form one hole in one shot by the Bessel beam but also the moderate etching selectivity exhibited by the borosilicate glass used in this study are great assets for rapidly fabricating large‐scale arrays that meet the demands for digital NAAT. Thus, the scheme presented in this article provides an important new application of ULISE using a Bessel beam. We also investigate the effects of fabrication parameters such as the pulse energy, pulse duration, and etching environment, for creating optimized hole profiles, and we discuss the possible underlying mechanism for laser processing. We further show the applicability of the developed technique to the fabrication of 3D ICs and the investigation of the fast motor control mechanisms for fruit flies.

## 2 Results

### 2.1 Laser Fabrication of Glass Through‐Hole Array

**Figure 1:**
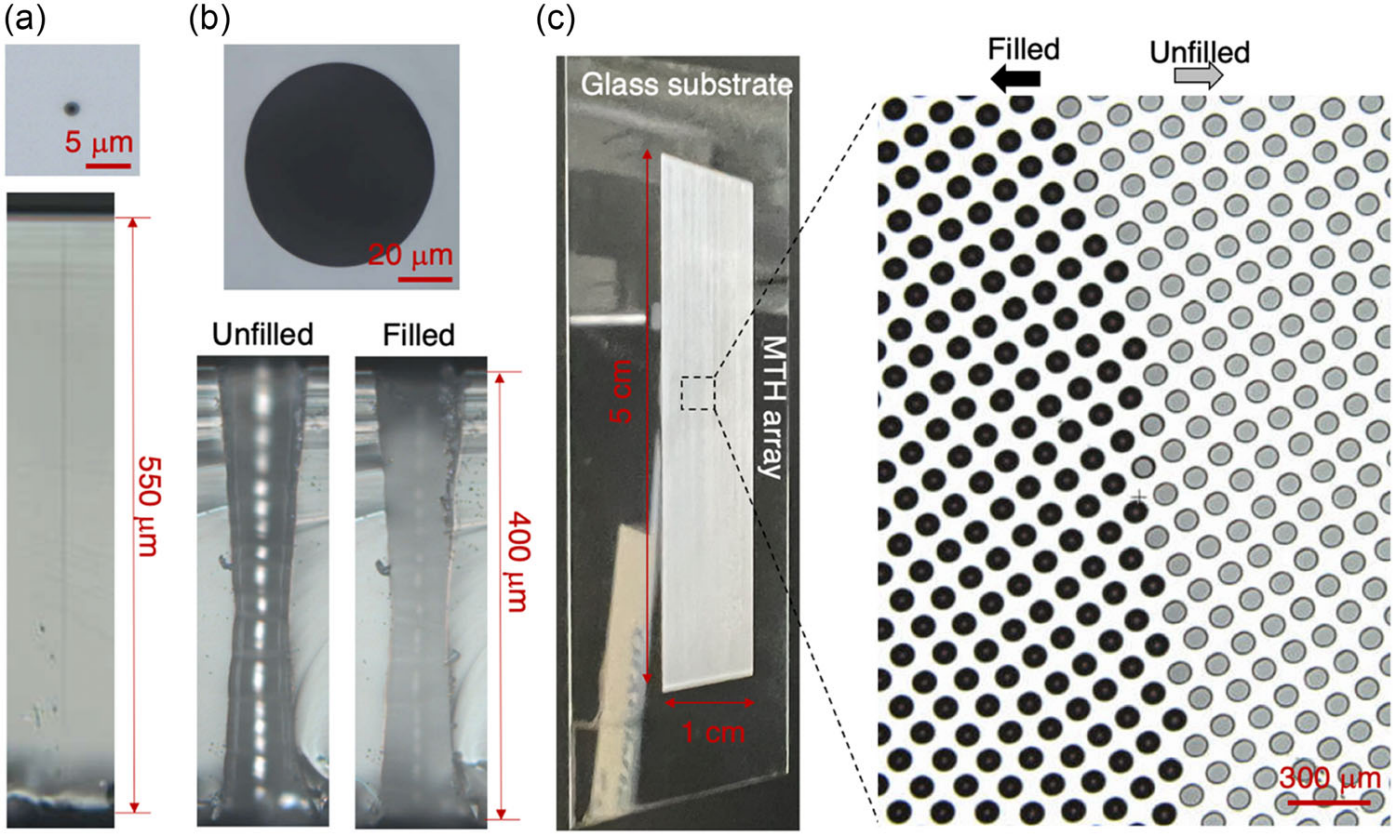
Fabrication of MTH array. a) Optical micrographs of glass modification immediately after single‐Bessel pulse irradiation (upper: surface, lower: cross section). b) Optical micrographs of MTH in glass substrate after chemical etching by hydrofluoric acid. (upper: surface, lower: cross section). The cross‐sectional images show both unfilled (left) and water‐filled (right) holes. c) Large‐scale MTH array in glass slide (left: photograph of ≈20 000MTH array, right: optical micrograph at boundary between filled and unfilled MTHs).

Bessel pulses (repetition rate of 100 Hz) were generated by passing an ultrafast laser through an axicon lens, two plano‐convex lenses, and a microscope objective lens and were then focused into a glass substrate that was translated continuously at 12 mm s^−1^ (see Methods). This led to the formation of 100 discrete LMR per second, where each LMR was created by a single Bessel pulse, with an interval of 120 μm along the scanning direction. The optical micrograph of the top surface of glass modified by a single Bessel pulse illustrates a central area of LMR that has a diameter of about 1 μm (**Figure** **1**a upper). The cross‐sectional image shows a uniformly wide trace line that extends from the top surface toward the bottom surface, suggesting that the Bessel beam has an effective depth of focus of ≈550 μm (extendable to 1 mm with higher pulse energy and proper modifications of optical setup), which matches and modifies the entire thickness of the glass substrate (Figure 1a lower). The glass substrate was then treated with a HF solution for selective etching. Although the etching time takes tens of minutes, which is significantly longer than the laser writing time (≈300 s for a 20 000‐spot array including time for acceleration and deceleration between each scanning line), etching is a batch process, allowing many samples to be processed simultaneously. The diameter of the created MTH is measured to be ≈70 μm at the surface (Figure 1b upper), which is much larger than that for the LMR. The cross‐sectional MTH images show a structure that is tapered from the top and bottom surfaces toward the center (Figure 1b lower). The large diameter and the tapered structure are due to the relatively low etching selectivity between the modified and unmodified regions. In addition, the total thickness of the glass substrate was reduced to ≈400 μm due to HF etching of the unmodified surfaces. Based on this thickness reduction, the etching selectivity ratio between the modified and unmodified regions is estimated to be around 4:1, which is moderate and suitable for creating the MTHs with medium aspect ratio (1:6) required for NAAT (an image showing a blind hole with low aspect ratio created with Gaussian beam is demonstrated in Figure S1, Supporting Information). To check the uniformity of created holes, the sizes of about 6000 MTHs on each of two glass chips with different hole sizes were measured (Figure S2, Supporting Information). The average hole diameter in the array represented by Figure S2a (Supporting Information) was calculated to be 78.2 μm with a root mean square error of 0.63 μm. Meanwhile, the average hole diameter in the array represented by Figure S2c (Supporting Information) was calculated to be 101.7 μm with a root mean square error of 1.4 μm. Thus, the uniformity of MTH size is high enough for the accuracy requirement of digital NAAT and can be further improved with advanced etching processing tools and using the glass substrate with better flatness. The measured dimensions give a volume of ≈3 nL for each MTH. Due to the hydrophilic nature of glass and capillary action (the fully penetrating nature of the MTHs ensures that liquid can easily flow from one side to the other), the MTHs can effectively overcome surface tension to trap liquid. To confirm this, an empty MTH (Figure 1b lower‐left labeled “Unfilled”) was filled by touching a droplet of water to one side of the empty hole. The water‐filled MTH appears brighter due to the higher refractive index of water than that of air (Figure 1b lower‐right labeled “Filled”). Overall, a ≈20 000 MTH array was fabricated during which laser pulse train continuously impinges on the glass slide with line‐by‐line scanning (Figure 1c left). Since the volume of a single MTH is estimated to be 3 nL, the whole array can thus hold a total reagent volume of 60 μL, which can be adjusted by changing the HF etching time. A portion of the MTH array was filled by dropping water onto the MTH array and then spreading it over the surface using a plastic blade, in which MTHs filled with water appear darker (Figure 1c right). Note that due to the suitable aspect ratio of the MTHs, the evaporation of internal liquid is slow, typically taking more than 15 min, providing enough time for subsequent processing steps such as chip sealing for thermocycling.

**Figure 2:**
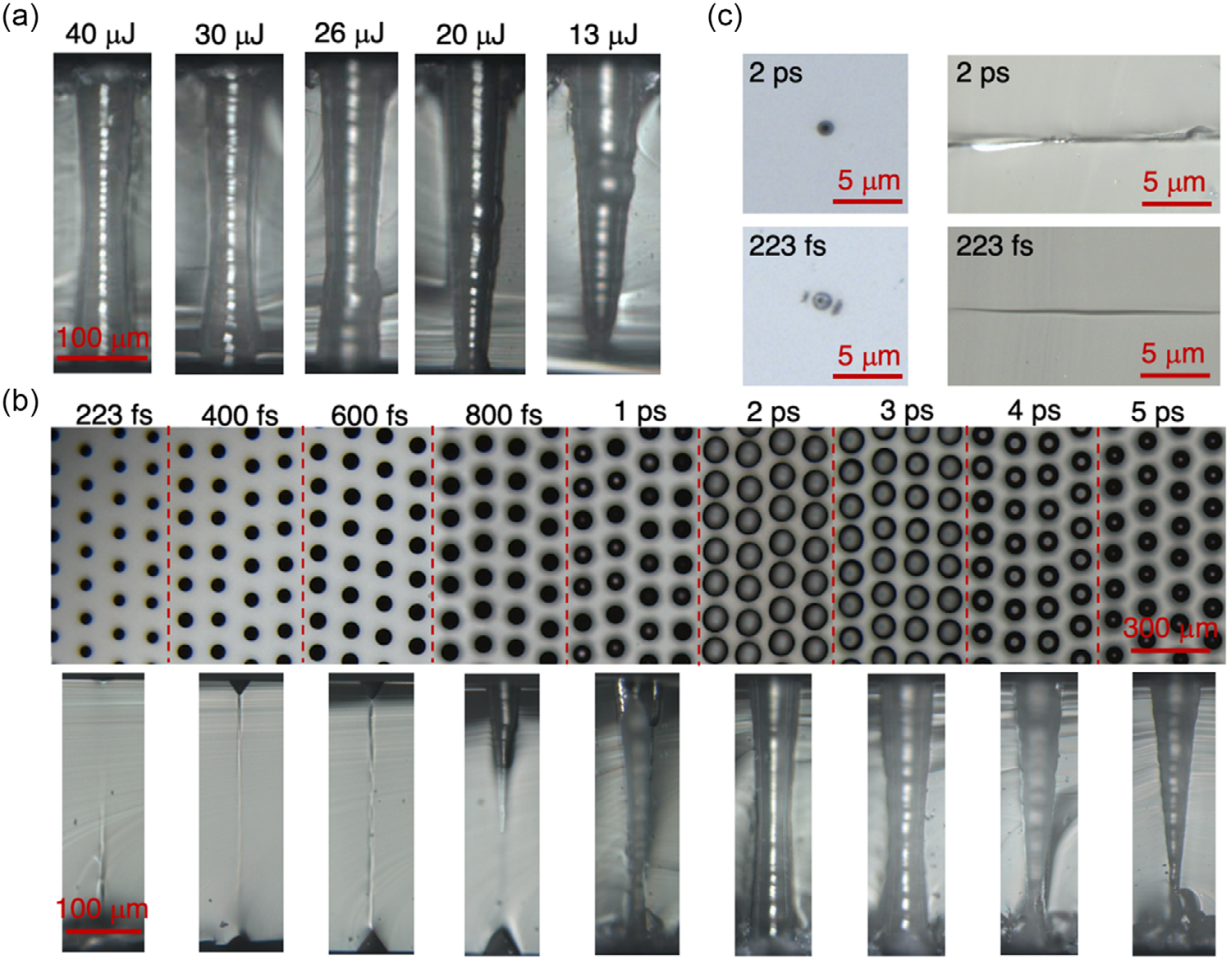
a) Cross‐sectional optical micrographs of MTHs fabricated at different pulse energies by ultrafast Bessel pulse processing and HF etching. b) Top‐view (upper) and side‐view (lower) images of MTHs fabricated using pulse durations from 223 fs to 5 ps (from left to right) by ultrafast Bessel pulse processing and HF etching. c) Top‐view (left) and side‐view (right) images of LMR following single Bessel pulse with duration of 2 ps and 223 fs before HF etching.

We next investigated the effects of laser parameters on MTH formation. First, laser pulse energies from 40 to 30 μJ, 26, 20, and 13 μJ were used to modify glass by a single Bessel pulse. The cross‐sectional optical micrographs of fabricated MTHs are demonstrated (**Figure** **2**a). It can be seen that for pulse energies of 26 μJ or larger, the MTHs have a relatively uniform diameter along the vertical direction (direction along the beam axis). However, for 20 and 13 μJ, the shape becomes significantly tapered toward the bottom surface. This suggests that the degree of modification has a gradient from the top surface to the bottom of glass for the pulse energy of 20 μJ and smaller. Also, since similar structures are obtained for a pulse energy of 26 μJ or larger, it appears that an energy threshold exists, above which the etching selectivity is constant, while below which the etching selectivity decreases (20 and 13 μJ). The tapered structures formed at lower pulse energies are probably due to multiphoton absorption of side lobes of the Bessel beam. Specifically, some of the energy in the side lobes is absorbed by multiphoton absorption by the glass during propagation, and the energy loss becomes larger with increasing depth. As a result, the intensity of central lobe in deeper regions is insufficient to modify the glass when the incident pulse energy is smaller.

The effect of the pulse duration on MTH formation was further investigated. The surface image of ULISE‐treated glass slide is illustrated in Figure 2b (upper row). Here all pulse energies were kept the same as 40 μJ. It is seen that MTH formation is less effective for pulse durations other than 2 or 3 ps in terms of sizes. Clearer views can be found in Figure 2b (lower row), which illustrates the cross‐sectional images of MTHs fabricated with the same pulse durations as those for the upper row. It can be clearly seen that pulse durations of 2 and 3 ps can result in good MTH formation, while durations of 4 and 5 ps only produce tapered holes. This may be explained by a decrease in the peak pulse energy for longer pulse durations, which may be below the modification threshold. As described above, some of the energy in the side lobes is wasted by multiphoton absorption during the propagation. This would reduce the depth of modified regions, in the case of longer pulses. On the other hand, for shorter pulses in the femtosecond regime, the etching selectivity is significantly reduced. MTHs cannot be formed for pulse durations of less than 1 ps, and there is almost no selective etching effect below 800 fs except near both surfaces of the glass slide. To further clarify the mechanism involved, LMRs produced by two different pulse durations of 2 ps and 223 fs at the same pulse energy of 40 μJ before HF etching are compared (Figure 2c). In the top‐view images of glass surfaces (left), 2 ps laser‐modified spot shows a darker central area than that created by 223 fs pulse. This may be attributed to thermal effects associated with the longer pulse. For the 223 fs pulse, surface modification by the side lobes of the Bessel beam can be seen. This is due to the higher side‐lobe intensity than for the 2 ps pulse and indicates that a significant portion of the side‐lobe energy is absorbed at the surface. In this situation, the energy remaining in the central lobe as it penetrates the glass may be below the modification threshold. From the cross‐sectional images (right), it is clear that the 2 ps pulse produces wider structural damage than the 223 fs pulse because of thermal effects and the larger energy of the central lobe inside the glass in the case of the longer pulse. The gradual increase of hole diameter with increasing pulse duration seen in Figure 2b (upper raw) may be also due to thermal effects and diffusion which create a wider modified region. The selective etching near the rear surface for femtosecond pulses (Figure 2b lower row) is likely due to the lower modification threshold at the surface than that in bulk.^[^ ^23^ ^]^ Thus, it can be concluded that the pulse duration is an important factor for creating high‐quality MTHs, with the optimum duration being 2 and 3 ps in this case. Finally, it is noted that the concentration of the HF solution used for etching has negligible effects on the MTH shape.

### 2.2 Digital NAAT Experiments

**Figure 3:**
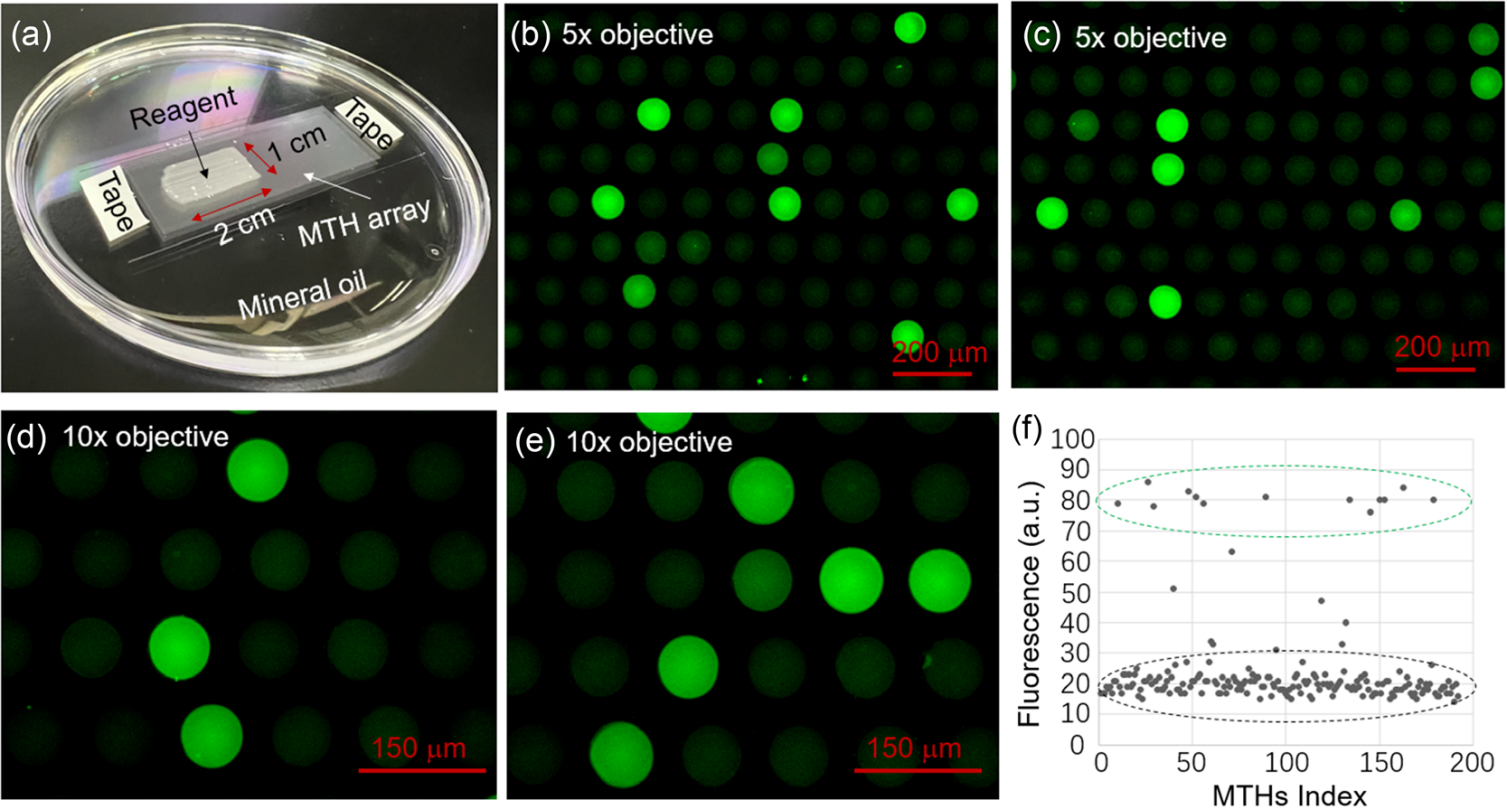
Digital NAAT experiment using glass MTH array. a) Photograph of MTH array chip filled with reagent containing DNA molecules for digital RPA. b,c) Surface optical micrographs (5× objective lens) from two different positions in MTH array chip after amplification process. d,e) Surface optical micrographs (10× objective lens) from two different positions in MTH array chip after amplification process. f) Scattering plots of average fluorescence intensity of each MTH in Figure 3b,c.

The digital NAAT performance of the developed MTH chips was tested in a proof‐of‐principle experiment using RPA, which was recently developed as a near‐room‐temperature amplification process without the need for thermal cycling.^[^ ^24^ ^]^ Briefly, RPA utilizes recombinases to insert primes into cognate sites by strand displacement activity. In contrast to the PCR process, which usually requires high‐temperature thermal treatment for denaturation and annealing, RPA can be conducted between 37 and 42 °C and thus may not require strict reagent sealing. It is important to point out that since the amplification is performed near room temperature, the process is initiated soon after target DNA molecules are mixed with the amplification reagent, so that a short partitioning process is required for digital RPA. For the test, around 10 000 MTHs (1 cm × 2 cm) were filled with 30 μL of reagent (**Figure** **3**a), which took less than 10 s. We mention that right after the loading process, the small amount of reagent remaining on the top surface area outside the MTHs was wasted reagent. However, such surface leftover was usually found to evaporate within several seconds, which is much shorter than the retention time in the MTHs (over 15 min) and may be considered negligible.

After the amplification process, fluorescence microscopy images were obtained at random locations on the MTH array using either a 5× or 10× objective lens (contrast enhanced by image processing) (Figure 3b–e). Enhanced fluorescence signals can be observed from several independent MTHs, which are associated with fluorescein amidites (FAM) dyes released during the DNA amplification process, indicating the presence of DNA molecules in these holes after the loading process. Fluorescence images of ≈1800 MTHs filled with reagent on a single glass chip after RPA, whose number is comparable to that used for other published digital PCR experiments,^[^ ^8^, ^25^ ^]^ were observed (Figure S3, Supporting Information). 116 MTHs out of ≈1800 MTHs showed fluorescence signals, indicating that the ratio of holes containing the DNA molecules was 6.4%. This ratio is very close to the expected value of 6% (=600/10.000: the average number of target DNA molecules per partition in this experiment). The small difference can be caused by incomplete sampling of the whole chip due to limitation of our instrument. Scattering plots of fluorescence intensity of MTHs in Figure 3b,c were created (Figure 3f) in which the *x* and *y* axes represent each individual MTH and the average fluorescence intensity of the corresponding MTH, respectively (after subtracting the background signal). It's clear that with few exceptional points, most of the MTHs with DNA molecule show fluorescence intensity around 80 (a.u.) (dashed green circles), which can be clearly distinguished with those without DNA molecule (intensity around 20 (a.u.), dashed black circles). Besides, in digital NAAT, it is important that the reagent partition units are independent of each other to ensure a correct statistical analysis. From the images, it can be seen that there exist single MTHs with enhanced fluorescence that are surrounded by adjacent MTHs without enhanced fluorescence, establishing that there is no connection between the MTHs.

As the DNA molecules obey a Poisson distribution when loaded on the chip, the probability of one MTH containing k DNA molecules can be calculated to be $\frac{\lambda^{k}e^{-\lambda }}{k!}$ = (94.18% for *k* = 0, 5.65% for *k* = 1, and 0.17% for *k* = 2), where λ is the average number of target DNA molecules per partition, which is 0.06 for this experiment.^[^ ^26^ ^]^ It can be seen that MTHs containing 0 or 1 DNA molecule account for over 99.83% of all loaded MTHs, meaning that most of the MTHs exhibiting enhanced fluorescence contain only 1 DNA molecule, which meets the requirement for absolute DNA quantification in digital NAAT.

### 2.3 Versatility of Ultrafast Bessel Beam Processing

**Figure 4:**
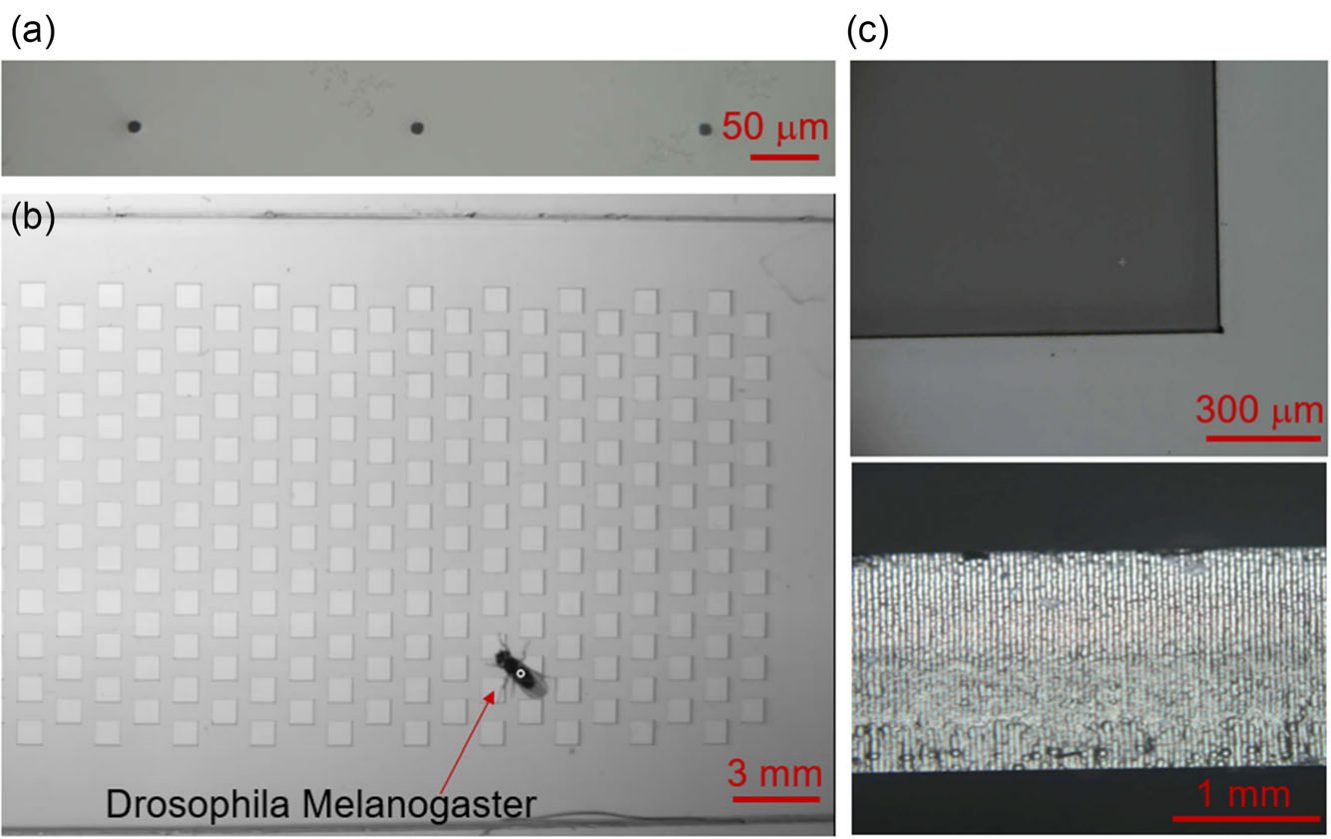
Versatile applications of ultrafast Bessel beam processing. a) High‐aspect‐ratio through‐holes in fused silica. b) Array of large square holes created by ultrafast laser cutting using Bessel beam and chemical etching for experiments with fruit flies (*Drosophila melanogaster*). c) Enlarged edge of square hole from top‐ and cross‐sectional view.

Finally, we briefly describe the multifunctionality of our laser fabrication technique using ultrafast Bessel pulses. For MTH array chips for NAAT, a medium‐hole‐aspect ratio (around 6:1) is preferable to efficiently load reagents. For other applications such as 3D IC packaging, glass through‐vias with a high aspect ratio are required to interconnect vertically stacked IC chips. In this case, the etchant should be replaced with KOH, because it provides much greater etching selectivity between modified and unmodified regions.^[^ ^27^ ^]^ Using the laser processing technique proposed in the present study followed by KOH etching, nontapered through‐holes with a diameter of 8 μm and an aspect ratio of ≈70:1 can be created in a fused silica substrate (**Figure** **4**a), which fulfils the requirement for future 3D ICs. In addition, high‐quality glass cutting with flexible shapes can be realized with sample translation under high‐repetition‐rate Bessel‐pulse irradiation followed by chemical etching, which has many possible applications. One such application is fabrication of an array of large square holes (0.5 mm) in a glass slide (Figure 4b, Figure S4, Supporting Information) for use in experiments with fruit flies (Drosophila Melanogaster). The fabricated chip can be used to study the fast motor control mechanisms that the fly implements for feet placement when it walks over the array of holes. The crack‐free, burr‐free capability of our technique (Figure 4c) is highly suitable for this kind of study since electrostatic forces caused by roughness at the hole edges are eliminated.^[^ ^28^ ^]^

## 3 Discussion

Rapid fabrication of large‐scale MTH arrays in glass substrates using ultrafast Bessel pulses has been demonstrated. The laser parameters were optimized to fabricate the MTH array chips with the quality and dimensions required for NAAT. A single Bessel pulse enabled formation of a single MTH, and it was possible to produce 20 000 MTAs at a writing speed of 100 MTHs per second. The fabrication speed can be further improved to more than 1000 MTHs per second by increasing the repetition rate and the translation speed using more advanced scanning systems. The MTH array chip was then tested in a digital NAAT experiment based on RPA and showed successful reagent partitioning. We should note that although RPA has obvious advantages, it has certain limitations such as nonspecific amplification under suboptimal conditions. Therefore, PCR is currently still the dominant NAAT, so that glass‐based chips are superior to those using other materials such as polymers due to the higher thermal conductivity of glass. The combination of ultrafast Bessel pulses and borosilicate glass with moderate etching selectivity offers rapid manufacturing of high‐quality MTH array chips for NAAT. The glass chip‐based reagent partitioning scheme achieved with ULISE can be an important route for highly efficient and low‐cost digital NAAT for future point‐of‐care diagnostics. ULISE is also expected to be adopted for a variety of other applications including glass through‐via fabrication for 3D IC packaging and flexible glass cutting.

## 4 Experimental Section

4.1

4.1.1

##### Laser Fabrication Setup

Laser processing was conducted using an ultrafast laser source (Light Conversion, Carbide), with an output wavelength of 1030 nm, a maximum pulse energy of 80 μJ, a tunable pulse duration from 223 fs to 20 ps, and a tunable repetition rate up to 1 MHz (Figure S5, Supporting Information). The power of the laser beam (diameter ≈1.5 mm) was controlled by a combination of a half waveplate and a polarization beam splitter. The laser was incident on an axicon lens with physical angle of *α* = 5° to generate the Bessel beam, which was then projected by a combination of two plano‐convex lenses and a 20× objective lens. Lens 1 had a focal length of 150 mm and was at a distance of 75 mm from the axicon lens, while lens 2 had a focal length of 125 mm and was at a distance of 35 mm from lens 1. The output of the laser beam from lens 2 was reflected by a dichroic mirror and entered an objective lens (Mitutoyo, M‐PLAN NIR 20X), which was 130 mm away from lens 2. The two planoconvex lenses were used to ensure that the whole beam diameter was smaller than the entrance pupil of the objective lens that demagnified the Bessel beam to achieve a high energy density for modification of glass. One advantage of this optical system is that it can utilize energy more efficiently by keeping the depth of focus as short as possible to match the thickness of the glass. The final output was focused into a borosilicate glass substrate with dimensions of 25 mm × 75 mm × 0.5 mm (Matsunami). For optimal laser processing, the pulse energy was set to 40 μJ with a pulse width of 2 ps at a repetition rate of 100 Hz, and the sample was translated at a speed of 12 mm s^−1^, so that an array of discrete modification spots with a spacing of 120 μm, each of which was created by a single of Bessel pulse, was formed in the glass substrate (Supplementary video). The creation rate for the modification spots was the same as the laser repetition rate.

##### Glass Etching

After laser processing, MTHs were created by hydrofluoric acid (HF) etching. The HF solution was purchased with a volume concentration of 46% (Fujifilm) and diluted to 10% with pure water. The glass substrate was placed inside the HF solution in a Teflon beaker, which was then placed in an ultrasonic cleaner filled with water at room temperature. The chemical batch process took 35 min, after which the glass substrate with formed MTHs were taken out and cleaned to remove residual HF (during HF etching, the diameter of a hole changed almost linearly with time: 10 min (28 μm), 20 min (50 μm), 30 min (75 μm), 40 min (100 μm)). For high‐aspect‐ratio through‐hole formation in fused silica, a similar etching process was applied using an 8 mol L^−1^ KOH solution (Fujifilm) with an etching time of 3 h at 80 °C. When the single shot of Bessel beam modifies the entire thickness of glass substrate, the final shape of a MTH is considered to be controlled by several factors. 1) Type of glass and concentration of HF solution, which determine the etching selectivity, and the higher etching selectivity, the smaller the diameter and thereby the higher the aspect ratio. 2) Etching time during which HF also induces isotropic etching of nonmodified glass, and the longer the etching time, the larger the diameter and the thinner the glass substrate resulting in the decrease of aspect ratio.

##### RPA Experimental Procedure

The reagents used were from the TwistAmp Basic Kit (TwistDx). We prepared a reaction mixture consisting of 8 μL of positive control primer mix, 29.5 μL of rehydration buffer, 1μL of positive control DNA template, and 9μL of water. We then added the reaction mixture to the TwistAmp basic reaction with 2.5 μL of 280 mM magnesium acetate. A volume of 30 μL of the final mixture of RPA reagent containing around 600 template DNA molecules was dropped onto the surface of the MTH array, and then a plastic blade was applied to press the reagent firmly against the surface and slide it from left to right, causing the reagent to be partitioned into each MTH (Figure S6, Supporting Information). Finally, both the top and bottom surfaces of the loaded chip were covered with mineral oil and the chip was kept at 40 °C for 25 min for amplification.

##### Microscopic Observation

After the RPA amplification process, the chip was observed under a fluorescence microscope (Model: Olympus BX51). The microscope was equipped with a 130 W fluorescence light source with an emission wavelength of 350–750 nm (Model: U‐HGLGPS). The emitted light was then guided to an excitation filter (transmission wavelength: 460–490 nm), reflected by a long‐pass dichroic mirror (cuton wavelength: 500 nm), and was passed through the objective lens and focused onto the sample. The FAM dyes in the MTHs were excited and emitted fluorescence at a wavelength of ≈520 nm, which travelled back through the objective, the dichroic mirror, and an emission filter (transmission wavelength: 520 nm and longer). The optical signal was finally collected and recorded by an optical camera.

## Conflict of Interest

The authors declare no conflict of interest.

## Author Contributions

J.Z. and K.S. designed the research. J.Z., K.O., and K.O. conducted laser processing research. J.Z., T.U., and Y.I. designed and performed digital nucleic acid amplification technique experiments. J.Z. and K.S. wrote the article.

## Supporting information

Supplementary Material

## Data Availability

The data that support the findings of this study are available from the corresponding author upon reasonable request.

## References

[1] P. T. Monis , S. Giglio , Infect. Genet. Evol. 2006, 6, 2. 16169776 10.1016/j.meegid.2005.08.004 PMC7106022

[2] T. Kang , J. Lu , T. Yu , Y. Long , G. Liu , Biosens. Bioelectron. 2022, 206, 114109. 35245867 10.1016/j.bios.2022.114109

[3] M. Teymouri , S. Mollazadeh , H. Mortazavi , Z. N. Ghale‐Noie , V. Keyvani , F. Aghababaei , M. R. Hamblin , G. Abbaszadeh‐Goudarzi , H. Pourghadamyari , S. M. R. Hashemian , H. Mirzaei , Pathol. Res. Pract. 2021, 221, 153443. 33930607 10.1016/j.prp.2021.153443 PMC8045416

[4] B. B. Oliveira , B. Veigas , P. V. Baptista , Front. Sens. 2, 2021, https://www.frontiersin.org/articles/10.3389/fsens.2021.752600

[5] R. Nyaruaba , C. Mwaliko , D. Dobnik , P. Neužil , P. Amoth , M. Mwau , J. Yu , H. Yang , H. Wei , Clin. Microbiol. Rev. 2022, 35, e00168‐21. 35258315 10.1128/cmr.00168-21 PMC9491181

[6] P.‐L. Quan , M. Sauzade , E. Brouzes , Sensors 2018, 18, 1271. 29677144 10.3390/s18041271 PMC5948698

[7] P. Zhu , L. Wang , Lab Chip 2017, 17, 34. 10.1039/c6lc01018k 27841886

[8] C. D. Ahrberg , J. W. Choi , J. M. Lee , K. G. Lee , S. J. Lee , A. Manz , B. G. Chung , Lab Chip 2020, 20, 3560. 32844858 10.1039/d0lc00788a

[9] H. Yin , Z. Wu , N. Shi , Y. Qi , X. Jian , L. Zhou , Y. Tong , Z. Cheng , J. Zhao , H. Mao , Biosens. Bioelectron. 2021, 188, 113282. 34020234 10.1016/j.bios.2021.113282 PMC8093165

[10] D. Conte , C. Verri , C. Borzi , P. Suatoni , U. Pastorino , G. Sozzi , O. Fortunato , BMC Genom. 2015, 16, 849. 10.1186/s12864-015-2097-9 PMC4619272 26493562

[11] S. Aralekallu , R. Boddula , V. Singh , Mater. Des. 2023, 225, 111517.

[12] F. Sima , J. Xu , D. Wu , K. Sugioka , Micromachines 2017, 8, 10.3390/mi8020040.

[13] L. A. Hof , J. Abou Ziki , Micromachines 2017, 8, 53.

[14] M. Hermans , J. Laser Micro Nanoeng. 2014, 9, 126.

[15] Y. Ding , L. Liu , C. Wang , C. Li , N. Lin , S. Niu , Z. Han , J. Duan , ACS Appl. Mater. Interfaces 2023, 15, 30985. 37315329 10.1021/acsami.3c04170

[16] C. Wang , K. Ding , Y. Song , X. Jia , N. Lin , J. Duan , Opt. Laser Technol. 2024, 168, 109829.

[17] S. Matsuo , H. Sumi , S. Kiyama , T. Tomita , S. Hashimoto , Appl. Surf. Sci. 2009, 255, 9758.

[18] D. Bischof , M. Kahl , M. Michler , Opt. Mater. Express 2021, 11, 1185.

[19] D. Wu , S.‐Z. Wu , J. Xu , L.‐G. Niu , K. Midorikawa , K. Sugioka , Laser Photonics Rev. 2014, 8, 458.

[20] L. Chen , D. Yu , J. Mater. Sci. Mater. Electron. 2021, 32, 16481.

[21] J. Kim , S. Kim , B. Kim , J. Choi , S. Ahn , Micromachines 2023, 14, 10.3390/mi14091766. PMC10536211 37763929

[22] Z. Wang , L. Jiang , X. Li , A. Wang , Z. Yao , K. Zhang , Y. Lu , Opt. Lett. 2018, 43, 98. 29328212 10.1364/OL.43.000098

[23] M. Chanal , V. Y. Fedorov , M. Chambonneau , R. Clady , S. Tzortzakis , D. Grojo , Nat. Commun. 2017, 8, 10.1038/s41467-017-00907-8. PMC5626724 28974678

[24] I. M. Lobato , C. K. O’Sullivan , TrAC Trends Anal. Chem. 2018, 98, 19. 10.1016/j.trac.2017.10.015 PMC7112910 32287544

[25] Z. Wu , Y. Bai , Z. Cheng , F. Liu , P. Wang , D. Yang , G. Li , Q. Jin , H. Mao , J. Zhao , Biosens. Bioelectron. 2017, 96, 339. 28525852 10.1016/j.bios.2017.05.021

[26] A. S. Basu , SLAS Technol. 2017, 22, 369. 28448765 10.1177/2472630317705680

[27] S. Kiyama , S. Matsuo , S. Hashimoto , Y. Morihira , J. Phys. Chem. C 2009, 113, 11560.

[28] T. Fujiwara , M. Brotas , M. E. Chiappe , Neuron 2022, 110, 2124. 35525243 10.1016/j.neuron.2022.04.008 PMC9275417

