# Supplementary material for: Rapid Manufacturing of Glass‐Based Digital Nucleic Acid Amplification Chips by Ultrafast Bessel Pulses
Source: Small Sci. 2023 Dec 28;4(2):2300166. doi: 10.1002/smsc.202300166 (PMC11935117; doi:10.1002/smsc.202300166)
Supplement: Supplementary file 1 — Supplementary Material [file SMSC-4-2300166-s001.zip › smsc.202300166-sup-0001-suppdata-S1.pdf]

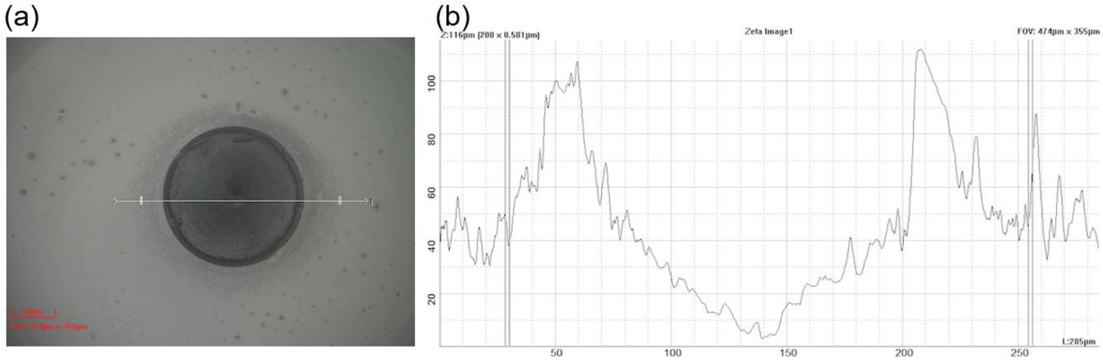

Fig. S1 – Glass hole fabricated by Gaussian beam followed with HF etching (a) Microscopic image of top surface after laser exposure and etching. (b) Cross section of the hole measure by an optical profiler, the hole is tapered and the deepest point is about 40 µm below the average height of surface.

As demonstrated in Fig. S2, around 6000 MTHs on two fabricated glass chips with different hole size separately to check their uniformity. Microscopic images were taken under an optical microscope with 5x magnification. Computer programming is used to automatically trace the edge of each hole (green circles) and calculate the radius. Calibration shows that 1 pixel represents 3 µm, based on which the averaged hole diameter of the first array is calculated to be 78.2 µm with root mean square error of 0.63 µm. Averaged hole diameter of the second array is calculated to be 101.7 µm with root mean square error of 1.4 µm.

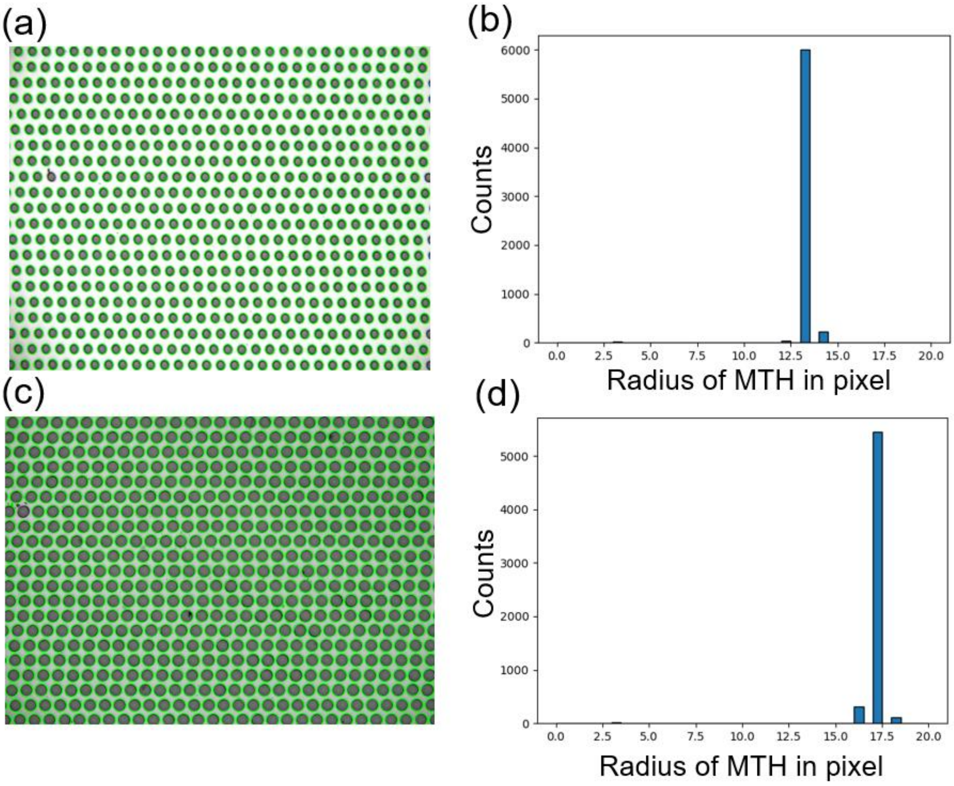

Fig. S2- Two MTH arrays were fabricated with different hole diameter. The diameter of around 6000 MTHs were measured on each array to check uniformity. (a) An representative microscopic image of the first array. (b) Histogram of the radii of MTHs of the first array. (c) An representative microscopic image of the second array. (d) Histogram of the radii of MTHs of the second array.

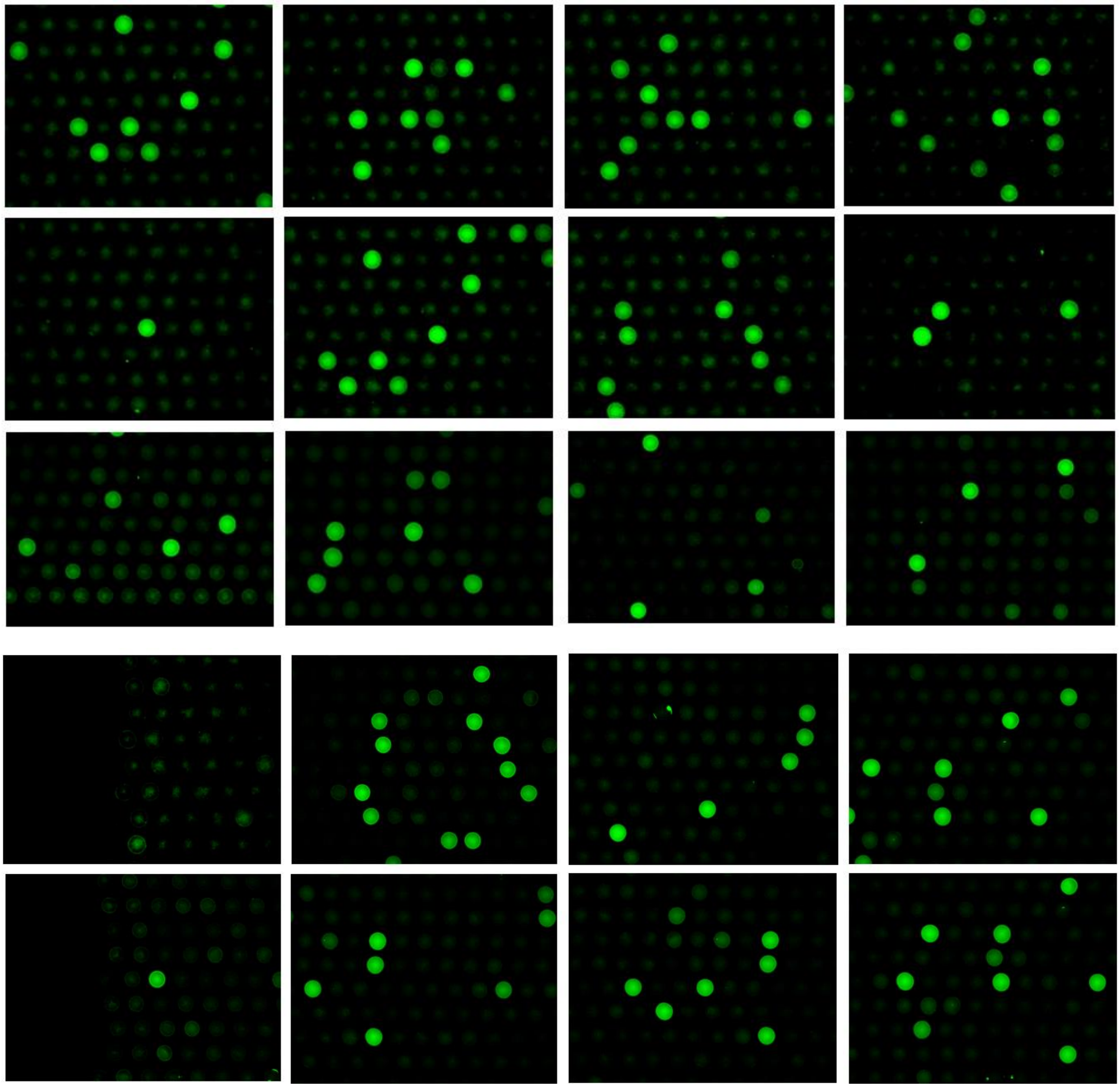

Fig. S3- 20 fluorescence images of the MTH array on a single glass chip after RPA. The images were taken at random locations by manually moving the MTH array chip under a fluorescence microscope (Olympus BX51). A total number of around 116 MTHs can be observed among totally ~1800 MTHs filled with reagents. The ratio is 6.4 %, very close to the expected value (6 %).

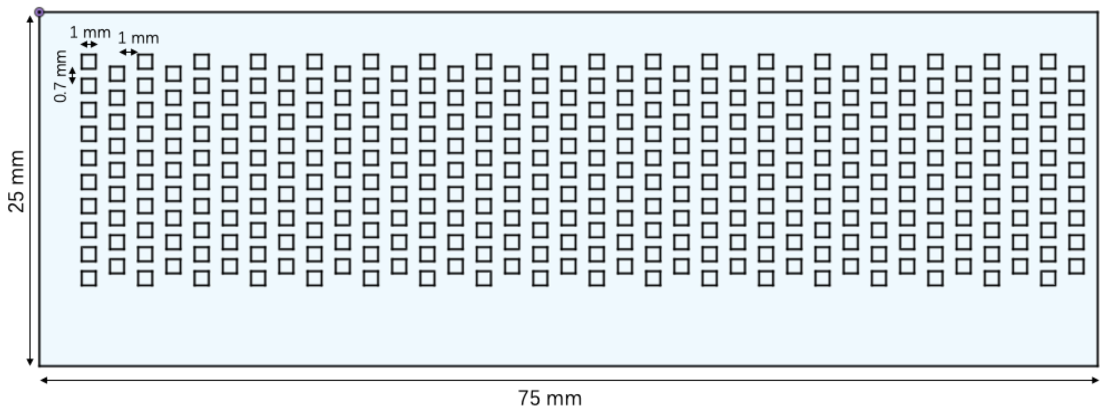

Fig. S4 – Schematic illustration of the large square hole array in a slide glass for observation of fruit fly walk.

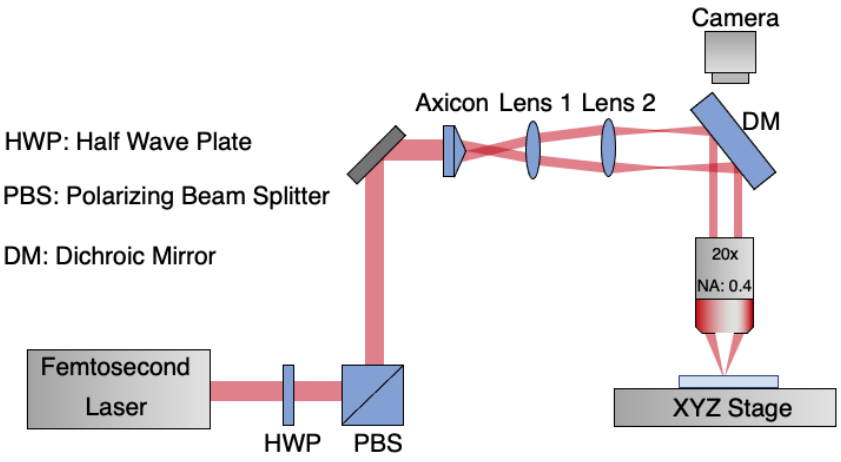

Fig. S5 – Optical setup for generation and demagnification of ultrafast Bessel pulses. Laser beam from the source is modulated in intensity with a half wave plate (HWP) and a polarizing beam splitter (PBS) cube and goes through multiple lenses to generate desired Bessel pulses, as described in detail in Methods. The final output is focused into the glass substrates on a translation stage.

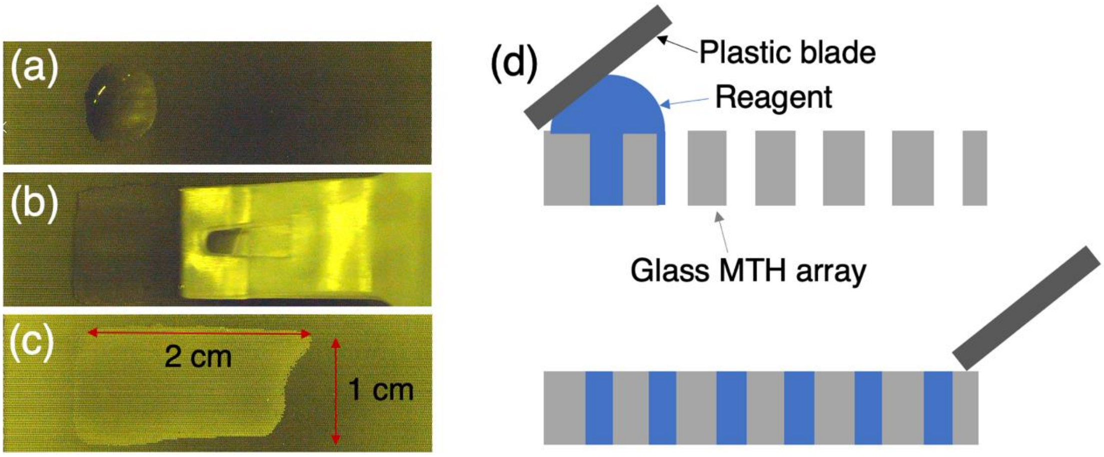

Fig. S6 – Procedure for reagent loading into a glass MTH array. (a) A droplet of reagent loaded on to the MTH array from a pipette. (b) A plastic blade used to slide the droplet into each holes. (c) The area of reagent filled MTH array, taken at one minute after step (b). (d) Schematic illustration of the reagent loading process from side view.
